# Scaling-up from an implementation trial to state-wide coverage: results from the preliminary Melbourne Diabetes Prevention Study

**DOI:** 10.1186/1745-6215-13-152

**Published:** 2012-08-28

**Authors:** Edward D Janus, James D Best, Nathalie Davis-Lameloise, Benjamin Philpot, Andrea Hernan, Catherine M Bennett, Sharleen O’Reilly, Rob Carter, Erkki Vartiainen, James A Dunbar

**Affiliations:** 1 Greater Green Triangle University Department of Rural Health, Flinders University and Deakin University, PO Box 423, Warrnambool, VIC, 3280, Australia; 2 Department of Medicine, North West Academic Centre, The University of Melbourne, Western Hospital, Footscray, VIC, 3011, Australia; 3 Melbourne Medical School, The University of Melbourne, Grattan Street, Parkville, VIC, 3010, Australia; 4 Faculty of Health, Deakin University, 221 Burwood Highway, Burwood, VIC, 3125, Australia; 5 Deakin Health Economics, Deakin Strategic Research Centre – Population Health, Faculty of Health, Deakin University, 221 Burwood Highway, Burwood, VIC, 3125, Australia; 6 National Institute for Health and Welfare, Mannerheimintie 166, 00300, Helsinki, Finland

**Keywords:** Type 2 diabetes, Prevention, Lifestyle, Intervention, Implementation, Randomised controlled trial, Effectiveness

## Abstract

**Background:**

The successful Greater Green Triangle Diabetes Prevention Program (GGT DPP), a small implementation trial, has been scaled-up to the Victorian state-wide ‘Life!’ programme with over 10,000 individuals enrolled. The Melbourne Diabetes Prevention Study (MDPS) is an evaluation of the translation from the GGT DPP to the Life! programme. We report results from the preliminary phase (pMDPS) of this evaluation.

**Methods:**

The pMDPS is a randomised controlled trial with 92 individuals aged 50 to 75 at high risk of developing type 2 diabetes randomised to Life! or usual care. Intervention consisted of six structured 90-minute group sessions: five fortnightly sessions and the final session at 8 months. Participants underwent anthropometric and laboratory tests at baseline and 12 months, and provided self-reported psychosocial, dietary, and physical activity measures. Intervention group participants additionally underwent these tests at 3 months. Paired *t* tests were used to analyse within-group changes over time. Chi-square tests were used to analyse differences between groups in goals met at 12 months. Differences between groups for changes over time were tested with generalised estimating equations and analysis of covariance.

**Results:**

Intervention participants significantly improved at 12 months in mean body mass index (−0.98 kg/m^2^, standard error (SE) = 0.26), weight (−2.65 kg, SE = 0.72), waist circumference (−7.45 cm, SE = 1.15), and systolic blood pressure (−3.18 mmHg, SE = 1.26), increased high-density lipoprotein-cholesterol (0.07 mmol/l, SE = 0.03), reduced energy from total (−2.00%, SE = 0.78) and saturated fat (−1.54%, SE = 0.41), and increased fibre intake (1.98 g/1,000 kcal energy, SE = 0.47). In controls, oral glucose at 2 hours deteriorated (0.59 mmol/l, SE = 0.27). Only waist circumference reduced significantly (−4.02 cm, SE = 0.95).

Intervention participants significantly outperformed controls over 12 months for body mass index and fibre intake. After baseline adjustment, they also showed greater weight loss and reduced saturated fat versus total energy intake.

At least 5% weight loss was achieved by 32% of intervention participants versus 0% controls.

**Conclusions:**

pMDPS results indicate that scaling-up from implementation trial to state-wide programme is possible. The system design for Life! was fit for purpose of scaling-up from efficacy to effectiveness.

**Trial registration:**

Australian and New Zealand Clinical Trials Registry ACTRN12609000507280

## Background

The Greater Green Triangle Diabetes Prevention Project (GGT DPP) was the first Australian implementation trial demonstrating that a lifestyle modification programme could be effectively implemented in a primary care setting, resulting in an estimated 40% diabetes risk reduction [1]. Following this success and a subsequent Healthy Living Course DPP randomised control trial [2], the Victorian government in 2007 announced funding for the Life! Taking Action on Diabetes (Life!) programme, a state-wide group-based lifestyle intervention targeting 25,000 Victorian residents aged over 50 at high risk of type 2 diabetes (T2DM) [3]. Programme goals are based on modifications to diet and physical activity. The Life! programme has direct lineage from the Finnish Diabetes Prevention Study [4], the Good Ageing in Lahti region Implementation trial [5] and the GGT DPP [1]. Over 10,000 people have enrolled in Life! courses, run by 208 group facilitators employed by 178 providers in Victoria (population 4,932,422 [6], area 237,629 km^2^).

Moving from trials to large-scale implementation has long been fraught with failure [7], so evaluation of the scaled-up programme is important to assess its outcome. The Melbourne Diabetes Prevention Study (MDPS) is an evaluation of the effectiveness and cost-effectiveness of the Life! programme while it is being rolled out state-wide. This evaluation is being undertaken to see the effect of scaling-up to state level from the small GGT DPP implementation trial.

The specific objectives of the MDPS are to evaluate a structured primary-care based diabetes prevention programme (Life!) being implemented in Victoria for people aged over 50 at high risk of developing T2DM by: monitoring clinical and behavioural outcomes before and after the intervention (particularly reduction in diabetes risk, weight loss and quality of life); comparing the Life! programme with another cohort receiving usual care; and undertaking an economic assessment of the Life! programme.

The MDPS was designed to recruit 1,300 individuals (650 intervention, 650 control) from a number of sources including primary healthcare practices and the general community. The simultaneous large-scale rollout of the Life! programme in the community meant that the Life! implementers had to address issues such as recruitment, changes to eligibility criteria, referral processes, structure and content, as well as alternative programme delivery for different population groups. This complicated our evaluation of the Life! programme. For these reasons the initial phase of the MDPS was converted to a preliminary pilot study (pMDPS). We present here the effectiveness results from the pMDPS. The information obtained from this pilot phase was then used to inform a subsequent larger MDPS that has now commenced.

## Methods

### Trial design

The pMDPS is a prospective, open, randomised controlled trial to assess effectiveness of a structured primary-care based diabetes prevention programme implemented in Victoria for people aged over 50 at high risk of developing T2DM. This is a parallel group study, with the intervention group receiving a diabetes prevention programme (Life!) and the control group receiving usual care.

### Participants

Individuals between 50 and 75 years at high T2DM risk were eligible to participate. High risk was defined as scoring 15 or above on the AUSDRISK tool, a 10-item questionnaire assessing T2DM risk [8]. Scores 15 to 19, and 20 and above respectively result in approximately one in seven and one in three developing T2DM within 5 years.

Exclusion criteria were diagnosed diabetes, cancer, severe mental illness, substance abuse, recent myocardial infarction, pregnancy, difficulty with spoken and written English, belonging to a cultural group for whom the AUSDRISK test is not calibrated [8] and other household members involved in study.

### Recruitment

**Figure 1:**
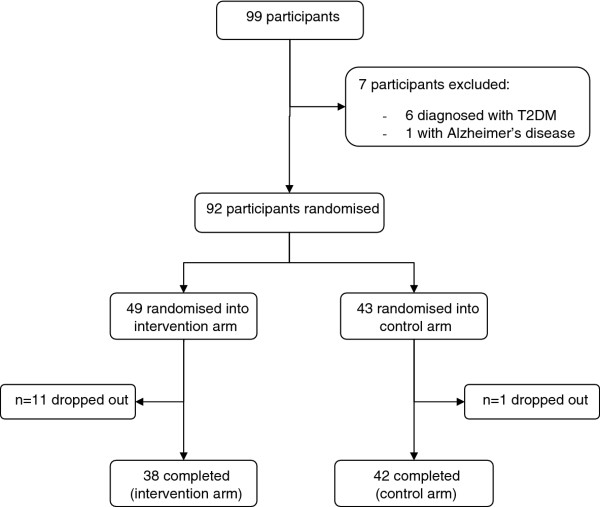
Participants in the preliminary Melbourne Diabetes Prevention Study.

During 2009 and 2010, 99 individuals were recruited from a number of sources including primary healthcare practices. Patients with impaired glucose tolerance or impaired fasting glucose were identified and contacted, and others were screened opportunistically in waiting rooms. Additional recruitment occurred at community events (see Figure 1).

### Informed consent

Informed consent was obtained in accordance with requirements of Deakin University Human Research Ethics Committee (Project Code 2009–066).

### Randomisation

pMDPS participants were individually randomised to an intervention (Life! programme) or control group. Randomisation was generated by a random number table and placed in individual sealed, opaque envelopes.

### Intervention

The intervention was a series of six structured group sessions. The first five sessions were at 2-week intervals and the final sixth session was 8 months after the first [1].

Control subjects continued with usual care provided by their general practitioner and were subsequently offered the Life! programme after 12 months.

Certified and accredited Life! facilitators (trained health professionals such as nurses or diabetes educators) delivered the intervention. A physiotherapist or exercise physiologist and a dietitian co-facilitated sessions three and four, respectively [9,10].

The Finnish Diabetes Prevention Study goals were used [4]: no more than 30% energy from fat; no more than 10% energy from saturated fat; at least 15 g/1,000 kcal fibre; at least 30 minutes/day moderate intensity physical activity; and at least 5% body weight reduction.

Processes and detailed goals for lifestyle change were individually tailored using a problem-solving and goal-setting approach.

### Measures

Participants in both groups underwent anthropometric and laboratory tests at baseline and 12 months (weight, height, body mass index, waist and hip circumference, blood pressure, triglycerides, total cholesterol, low-density lipoprotein-cholesterol, high-density lipoprotein-cholesterol, fasting plasma glucose, 2-hour oral glucose tolerance test (OGTT), and glycated haemoglobin). Intervention group participants additionally underwent these tests, except OGTT, at 3 months. Participants provided self-reported measures of depression and anxiety (Hospital Anxiety and Depression Scale) [11], and fat and fibre consumption (Food Frequency Questionnaire) [12]. The primary outcomes under investigation are changes in diabetes and CVD risk as determined by changes in weight, waist circumference, fasting plasma and 2-hour glucose, blood pressure and lipids. The secondary outcomes include changes in lifestyle behaviour and the Hospital Anxiety and Depression Scale depression score.

### Statistical methods

Statistical analyses were conducted in Stata 11.2 (College Station, Texas, United States) and PASW Statistics 18.0.3 (IBM SPSS. Armonk, New York, United States). Two-sided paired *t* tests were used to analyse within-group changes over time. Chi-square tests were used to analyse differences between groups in goals met at 12 months. Differences between groups for changes over 12 months were originally analysed using analysis of covariance, adjusted for baseline values. Subsequently, generalised estimating equations (GEE) were used to satisfy the intention-to-treat principle, including 3-month data (collected for intervention only) in the model. Quasi-likelihood under the independence model criterion was used to determine the best working correlation structure. *P* values (Table 1) are from GEE, and analysis of covariance results are presented in the text.

**Table 1:** Changes over time by treatment arm

	**Intervention**				**Control**			**Intervention vs. control**	
		**Baseline**	**Δ3 months**	**Δ12 months**		**Baseline**	**Δ12 months**	**Δ12 months**	
	***n***	**Mean (SD)**	**Mean (SE)**	**Mean (SE)**	***n***	**Mean (SD)**	**Mean (SE)**	**Mean (SE)**	***P*value**
BMI (kg/m^2^)	38	31.4 (4.82)	−0.86 (0.15)*	−0.98 (0.26)*	41	30.1 (4.19)	−0.21 (0.12)	−0.77 (0.29)	<0.001
Weight (kg)	38	87.2 (12.5)	−2.38 (0.40)*	−2.65 (0.72)*	41	81.8 (14.4)	−0.60 (0.33)	−2.05 (0.79)	0.844
Waist (cm)	38	106.5 (8.35)	−5.04 (1.00)*	−7.45 (1.15)*	41	101.7 (11.52)	−4.02 (0.95)*	−3.42 (1.48)	0.334
Hip (cm)	38	112.7 (9.24)	−1.97 (0.88)*	−3.18 (1.26)*	41	110.8 (9.90)	0.37 (1.05)	−3.56 (1.63)	0.348
SBP (mmHg)	38	135.9 (18.61)	−6.63 (2.25)*	−6.55 (2.39)*	41	132.1 (14.04)	−0.45 (2.86)	−6.10 (3.75)	0.126
DBP (mmHg)	38	80.1 (7.85)	−1.25 (1.31)	0.70 (1.55)	41	78.4 (7.84)	1.43 (1.75)	−0.73 (2.35)	0.963
TC (mmol/l)	37	4.97 (1.15)	−0.08 (0.10)	−0.09 (0.12)	39	5.05 (0.88)	0.06 (0.15)	−0.15 (0.19)	0.225
TG (mmol/l)	37	1.41 (0.50)	−0.07 (0.12)	−0.09 (0.08)	39	1.44 (0.64)	0.01 (0.08)	−0.10 (0.11)	0.227
HDL-cholesterol (mmol/l)	37	1.43 (0.35)	0.01 (0.03)	0.07 (0.03)*	39	1.59 (0.48)	−0.05 (0.04)	0.12 (0.05)	0.259
LDL-cholesterol (mmol/l)	37	2.89 (1.01)	0.11 (0.15)	−0.12 (0.11)	39	2.79 (0.84)	0.11 (0.12)	−0.23 (0.17)	0.207
FPG (mmol/l)	37	5.17 (0.44)	−0.07 (0.08)	−0.03 (0.06)	39	5.17 (0.44)	0.05 (0.07)	−0.08 (0.10)	0.219
2-hour OGTT (mmol/l)	36	6.64 (1.76)	n/a	−0.11 (0.30)	37	6.08 (1.50)	0.59 (0.27)*	−0.70 (0.40)	0.232
HbA1_c_ (%)	37	5.86 (0.30)	−0.05 (0.03)	0.08 (0.07)	39	5.89 (0.33)	0.13 (0.08)	−0.05 (0.11)	0.218
HADS-A	38	6.08 (3.35)	−0.53 (0.45)	−0.68 (0.38)	41	5.15 (3.18)	0.08 (0.34)	−0.76 (0.51)	0.467
HADS-D	38	3.16 (2.35)	0.03 (0.36)	0.18 (0.34)	41	2.90 (2.84)	0.02 (0.27)	0.17 (0.43)	0.876
Total fat (%)	33	36.3 (4.45)	n/a	−2.01 (0.83)*	37	36.2 (4.51)	−0.42 (0.77)	−1.59 (1.13)	0.290
Saturated fat (%)	33	14.6 (3.25)	n/a	−1.64 (0.51)*	37	14.0 (2.93)	0.29 (0.38)	−1.94 (0.62)	0.088
Fibre (g/day)	33	13.6 (2.97)	n/a	1.95 (0.58)*	37	13.5 (3.32)	0.51 (0.47)	1.45 (0.75)	0.030

Δ, change over time; SD, standard deviation; SE, standard error; BMI, body mass index; SBP, systolic blood pressure; DBP, diastolic blood pressure; TC, total cholesterol; TG, triglycerides; HDL, high-density lipoprotein; LDL, low-density lipoprotein; FPG, fasting plasma glucose; OGTT, oral glucose tolerance test glucose level; HbA1_c_, glycated haemoglobin; HADS-A, Hospital Anxiety and Depression Scale anxiety score; HADS-D, Hospital Anxiety and Depression Scale depression score; Total fat, percentage energy from total fat; saturated fat, percentage energy from saturated fat; fibre, grams of fibre per 1,000 kilocalories of energy; n/a, not measured at 3 months. *P <0.05 for within-group comparison.

Estimated diabetes risk reduction was based on waist circumference and weight change separately using results from two clinical trials [13-15]. In this study and in comparator trials, the mean difference between intervention and control as a percentage of the sample size-weighted baseline mean was calculated. Separate estimated risk reductions for this study were then found by assuming a simple linear relationship between percentage change and risk reduction in each of the comparator studies. The overall estimated diabetes risk reduction is the sample size-weighted mean of the previous two estimates.

## Results and discussion

After exclusion of seven individuals (six with newly diagnosed T2DM at baseline OGTT), 92 participants were randomised (*n* = 49 intervention, *n* = 43 control) – of whom 80 (*n* = 38 intervention, *n* = 42 control) completed the study (Figure 1). Information at 12 months was available for 38 intervention and 42 control participants. Baseline characteristics are shown in Table 2. The control group contained a higher proportion of women and lower-income individuals.

**Table 2:** Baseline characteristics of the participants

**Characteristic**	**Intervention**	**Control**
Overall	38 (47.5%)	42 (52.5%)
Gender		
Male	17 (44.7%)	10 (23.8%)
Female	21 (55.3%)	32 (76.2%)
Income		
Low	20 (54.1%)	29 (74.4%)
Medium	15 (40.5%)	9 (23.1%)
High	2 (5.4%)	1 (2.6%)
Aboriginal or Torres Strait Islander		
Yes	0 (0.0%)	0 (0.0%)
No	34 (100.0%)	38 (100.0%)
Age (years)	64.2 (7.5)	65.0 (6.0)
Education (years)	11.9 (3.2)	11.1 (2.9)

Data presented as *n* (%) or mean (standard deviation).

Main outcomes are shown in Table 1. Despite randomisation, differences between intervention and control subjects were observed in some baseline measures, especially weight, waist circumference and OGTT glucose at 2 hours.

The intervention group showed significant reductions at both 3 and 12 months in mean body mass index, weight, waist and hip circumferences and systolic blood pressure. Significant improvements were also seen at 12 months in high-density lipoprotein-cholesterol and all diet measures. In control subjects, OGTT glucose at 2 hours deteriorated and only waist circumference reduced significantly.

The intervention group significantly outperformed control subjects in changes over 12 months for body mass index reduction and fibre intake according to GEE results. After adjusting for baseline in analysis of covariance analyses, results were similar to the GEE estimates, except that the intervention group also showed greater weight loss and reduction in saturated fat intake (*P*= 0.035 and *P* = 0.003, respectively; data not shown). Although other differences were not significant, all favoured the intervention group, apart from Hospital Anxiety and Depression Scale depression score.

Table 3 shows goal attainment. Intervention participants were more likely to achieve at least one goal (*P* = 0.013). At least 5% weight loss was achieved by 32% of the intervention group compared with none of the controls. Few attained the physical activity goal. While there were favourable improvements in total and saturated fat and fibre consumption (Table 1) towards goals, only the fibre-intake goal (at least 15 g/1,000 kcal energy) was frequently achieved (Table 3).

**Table 3:** Preliminary Melbourne Diabetes Prevention Study goals at 12 months

**Goal**	***n***	**Intervention**	**Control**	***P*value**
<30% energy from total fat	70	7 (21.2)	5 (13.5)	0.394
<10% energy from saturated fat	70	4 (12.1)	2 (5.4)	n/a
>15 g fibre/1,000 kcal energy	70	19 (57.6)	14 (37.8)	0.099
≥30 minutes/day moderate-level PA	79	4 (10.8)	4 (9.5)	n/a
≥5% weight loss	79	12 (31.6)	0 (0.0)	<0.001
Number of goals	69			n/a
0		7 (21.2)	18 (50.0)	
1		15 (45.5)	12 (33.3)	
2		5 (15.2)	6 (16.7)	
3		5 (15.2)	0 (0.0)	
4		1 (3.0)	0 (0.0)	
5		0 (0.0)	0 (0.0)	

Data presented as *n* (%). PA, physical activity.

## Conclusions

This preliminary study shows that it is possible to scale-up from an implementation trial for diabetes prevention (GGT DPP) and from the Victorian-based Healthy Living Course DPP [2] to a state-wide programme (Life!) with comparable outcomes. The results are tending to show that the system design for Life! will be fit for the purpose of scaling-up to a state-wide programme [3], but we will have to wait for the final MDPS results to confirm this. Participants achieved an estimated 40% diabetes risk reduction based on a decrease in waist circumference and 24% based on weight reduction, an outcome comparable with GGT DPP [1] and better than the FIN-D2D Finnish National Diabetes Prevention Program [16] and the Good Ageing in Lahti region trial [5].

Better outcomes were seen in the intervention group for anthropometric and biochemical measures, although some were not statistically significant. Goal achievement was not as successful as for the GGT DPP [1], the Finnish Diabetes Prevention Study [4] and the Good Ageing in Lahti region trial [5].

At this stage, the small sample size has reduced the generalisability of our findings. Groups were not particularly well matched at baseline and 22% of the intervention group dropped out, which may have confounded the results. Measurement bias may also have contributed to the high waist circumference reduction. Valuable lessons about methodology, especially recruitment and quality control for anthropometric measurements, have been learnt. These have been addressed in the forthcoming MDPS, which has now commenced. One benefit of this study was early diagnosis of six new cases of T2DM during recruitment.

Implementation failure is commonplace [7] because trials often provide insufficient information to facilitate successful scale-up. Moderating variables and issues of generalisability are frequently unreported and external validity is untested or uncertain [17].

Specifically, the challenges of translating clinical trials into effective population programmes have been reported in the literature [18,19]. There has been a call to identify the barriers to diabetes prevention in the real world, with a special focus on identifying efficient intervention methods and delivery mechanisms [19]. Additionally, the importance of taking into account local circumstances and establishing partnerships and collaborations across sectors has been noted [18]. This has been achieved through the development of recommendations and guidelines for prevention of T2DM [18,19]. These frameworks should be useful in our evaluation processes as we continue with diabetes prevention in Australia and with our evaluation of the state-wide Life! programme.

## Abbreviations

GEE: generalised estimating equations; GGT DPP: Greater Green Triangle Diabetes Prevention Program; MDPS: Melbourne Diabetes Prevention Study; OGTT: oral glucose tolerance test; pMDPS: preliminary-phase Melbourne Diabetes Prevention Study; SE: standard error; T2DM, type 2 diabetes.

## Competing interests

The authors declare that they have no competing interests.

## Authors’ contribution

EDJ, JDB, RC, EV and JAD were responsible for the research question, designed the study, and were responsible for obtaining funding for the study. ND-L and AH wrote the first draft of this manuscript, and with EDJ were responsible for the revisions. CMB and SO’R contributed to specific sections of the manuscript. BP is the statistician and performed the power calculation, the sample size considerations, offered advice, and performed and wrote the statistical analysis, and with ND-L wrote the results sections. RC is the team’s expert in economic evaluation and was involved in the design of the study, and reviewing the manuscript. JAD is the general supervisor of the study and was involved in revising the article. All authors read and approved the final version of the manuscript.
